# Vicinal ketoesters – key intermediates in the total synthesis of natural products

**DOI:** 10.3762/bjoc.18.129

**Published:** 2022-09-15

**Authors:** Marc Paul Beller, Ulrich Koert

**Affiliations:** 1 Fachbereich Chemie, Philipps-Universität Marburg, Hans-Meerwein-Straße 4, 35032 Marburg, Germany

**Keywords:** aldol addition, ketoesters, natural products, total synthesis

## Abstract

This review summarizes examples for the application of vicinal ketoesters such as α-ketoesters, mesoxalic esters, and α,β-diketoesters as key intermediates in the total synthesis of natural products utilizing their electrophilic keto group as reactive site. Suitable key reactions are, e.g., aldol additions, carbonyl ene reactions, Mannich reactions, and additions of organometallic reagents. The vicinal arrangement of carbonyl groups allows the stabilization of reactive conformations by chelation or dipole control.

## Introduction

**Scheme 1:**
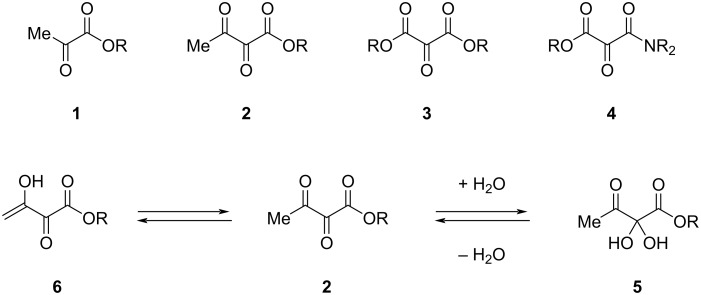
Structures of vicinal ketoesters and examples for their typical reactivity.

Vicinal ketoesters contain a carbonyl group adjacent to an ester group. One keto group results in α-ketoesters **1** and two vicinal keto groups lead to α,β*-*diketo esters **2** (Scheme 1). On the other hand, two carboxylic acid functionalities adjacent to a keto group result in mesoxalic diesters **3**, or mesoxalic ester amides **4**. The increased electrophilicity of the keto group and the high density of these complex functional groups make such structures attractive as key intermediates for the total synthesis of natural products [1]. Thus, the high electrophilicity of the central carbonyl group in α,β*-*diketoesters **2** allows the formation of stable hydrates **5**. In case of an enolizable position enolization (**2**→**6**) is facilitated.

**Scheme 2:**
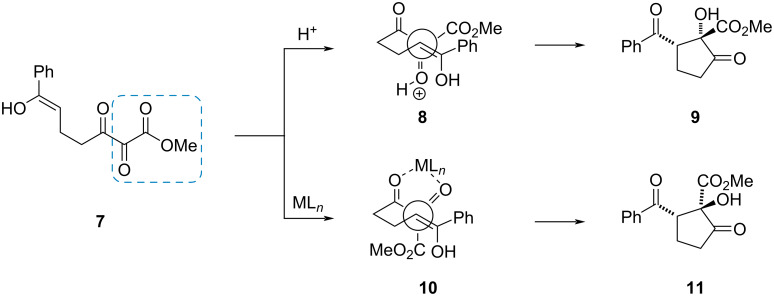
Doyle’s diastereoselective intramolecular aldol addition of α,β-diketoester.

The chemistry of vicinal polycarbonyl compounds such as *vic*-diketoesters has been investigated in depth by Wasserman, Parr [2] and Gleiter, Rubin [3]. Important contributions for the use of α,β-diketoesters in stereoselective transformations came from Doyle’s group [4–5]. One remarkable example is the diastereoselective intramolecular aldol addition of ketones such as **7** (Scheme 2) [5]. Brønsted-acid catalysis leads via a transition state **8** to the aldol **9**, while the use of chelating Lewis acids results via **10** in the epimeric aldol **11**.

This review is a collection of total syntheses of natural products where vicinal keto esters were used as key intermediates. For reasons of clarity and better comparability all syntheses are strongly summarized highlighting the key step only.

The presentation of the examples is structured in three parts:

1. **α-Ketoesters** as key intermediates: (+)-euphorikanin A, (−)-preussochromone A, (−)-preussochromone D, (−)-jiadifenoxolane A, palau’amine, jatrophen, (−)-hopeanol, (+)-campthotecin, isoretronecanol, corynoxine, (+)-gracilamine, (−)-irofulven.
2. **Mesoxalic** diester and ester amides as key intermediates: (+)-awajanomycin, (−)-aplaminal, cladoniamide G.
3. **α,β-Diketoesters** as key intermediates: preussochromones E and F.

## Review

### 1. α-Ketoesters as key intermediates:

#### (+)-Euphorikanin A

**Scheme 3:**
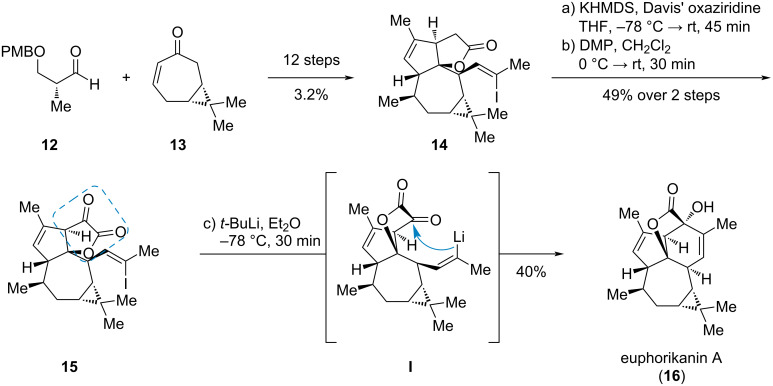
Synthesis of euphorikanin A (**16**) by intramolecular, nucleophilic addition [6].

In the final step of the synthesis of (+)-euphorikanin A (**16**), an ingenane-derived diterpenoid with a 5/6/7/3-fused tetracyclic carbon skeleton, Carreira et al. used an intramolecular nucleophilic addition of an alkenyl metal species to the α-ketoester **15** (Scheme 3) [6]. The ketoester **15** was synthesized by a chiral pool approach starting from (+)-3-carene derived cycloheptenone **13** [7–8] and aldehyde **12** (accessible from (*R*)-Roche ester [9]) via the γ-lactone **14**. The ketoester moiety was established by an enolate hydroxylation with Davis’ oxaziridine and subsequent oxidation using Dess–Martin periodinane. Initial attempts for the key step (**15** → **16**) like a Nozaki–Hiyama–Kishi reaction failed, but lithium–halogen exchange using *t-*BuLi at low temperatures gave the desired vinyllithium intermediate **I** which successfully added to the desired α-carbonyl group.

#### (−)-Preussochromone A

**Scheme 4:**
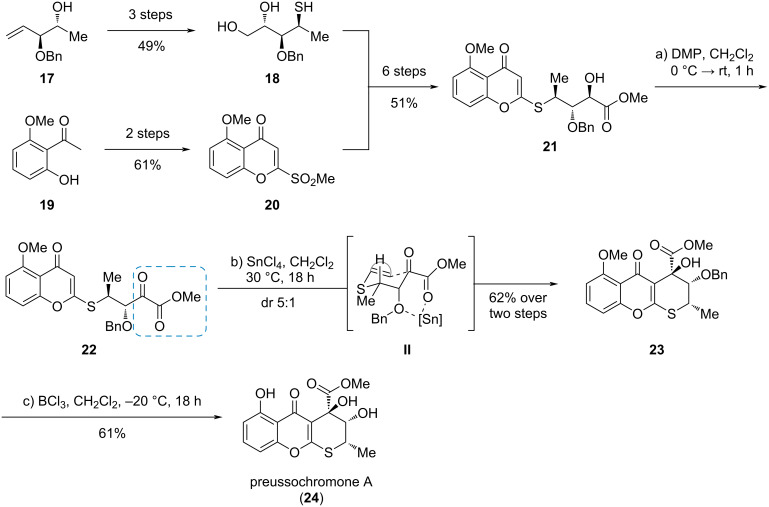
Ketoester cycloisomerization for the synthesis of preussochromone A (**24**) [10].

In 2020, the Koert group disclosed the synthesis of (−)-preussochromone A (**24**), a fungal metabolite with a highly substituted tetrahydrothiopyrane core annulated to a chromenone [10]. The tetrahydrothiopyrane ring was closed by a Lewis-acid-promoted cycloisomerization of the α-ketoester **22**, which can be described as a Friedel–Crafts-type reaction or an aldol reaction of an *S*,*O*-ketene acetal (Scheme 4). The required ketoester **22** was synthesized from sulfonylchromenone **20**, accessible from dihydroxyacetophenone **19** and thiol **18** derived from known alcohol **17** [11–12]. DMP oxidation of α-hydroxyester **21** and subsequent cycloisomerization led to the desired cyclization product **23** via transition state **II** in a dr of 5:1. Final deprotection gave preussochromone A (**24**).

#### (−)-Preussochromone D

**Scheme 5:**
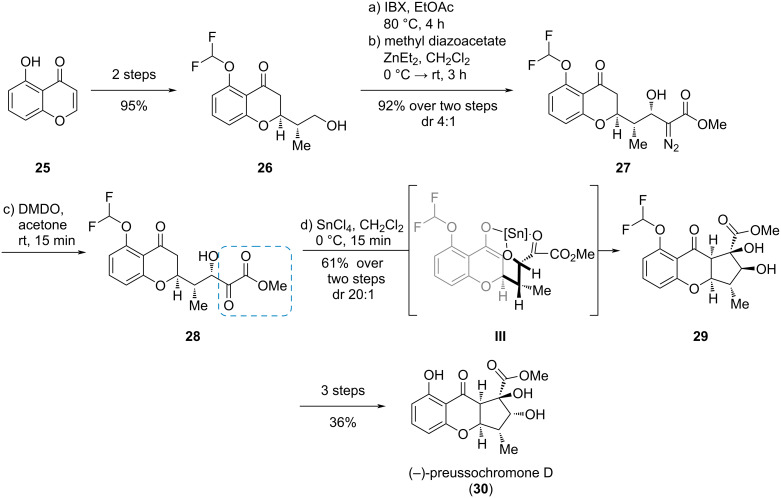
Diastereoselective, intramolecular aldol reaction of an α-ketoester **28** in the synthesis of (−)-preussochromone D (**30**) [13–14].

A similar approach was chosen in the synthesis of the structurally related natural product preussochromone D (**30**) reported by Koert et al. [13]. The synthesis commenced with the efficient production of alcohol **26** from 5-hydroxy-4*H*-chromen-4-one (**25**, Scheme 5) [14]. The ketoester moiety was build up via oxidation and nucleophilic addition of methyl diazoacetate, yielding alcohol **27**. Subsequent oxidation gave α-ketoester **28** which was used in an intramolecular, Lewis acid-mediated aldol reaction, presumably via tridentate complex transition state **III**, to give diol **29** as a single diastereomer. Inversion of the secondary alcohol and deprotection gave preussochromone D (**30**).

#### (−)-Jiadifenoxolane A

**Scheme 6:**
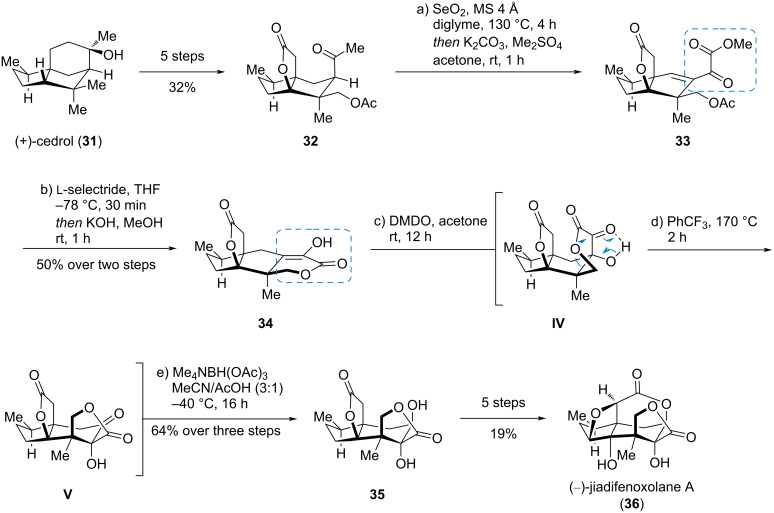
Synthesis of an α-ketoester through Riley oxidation and its use in an α-ketol rearrangement in the synthesis of (−)-jiadifenoxolane A (**36**) [15].

The *Illicium* sesquiterpenes containing a *seco*-prezizaane carbon framework are highly oxidized, structurally complex natural products. Maimone et al. published a remarkable synthesis of the *Illicium* sesquiterpene (−)-jiadifenoxolane A (**36**), starting from the abundant sesquiterpene (+)-cedrol (**31**, Scheme 6) [15]. Through a series of finely tuned CH oxidations, cedrol (**31**) was converted to the lactone **32**. In a single step, using Riley oxidation conditions, the methyl ketone moiety was transferred to the α-ketoester **33**. Reduction, lactonization, and elimination gave the ketoesters-derived enol **34**. Oxidation of the latter compound to the α-keto-β-hydroxy ester **IV** using DMDO and subsequent heating in PhCF_3_ triggered an α-ketol rearrangement which led to ketol **V**. Diastereoselective reduction gave α,β-dihydroxyester **35** which was converted to (−)-jiadifenoxolane A (**36**) in five further steps.

#### Palau’amine

**Scheme 7:**
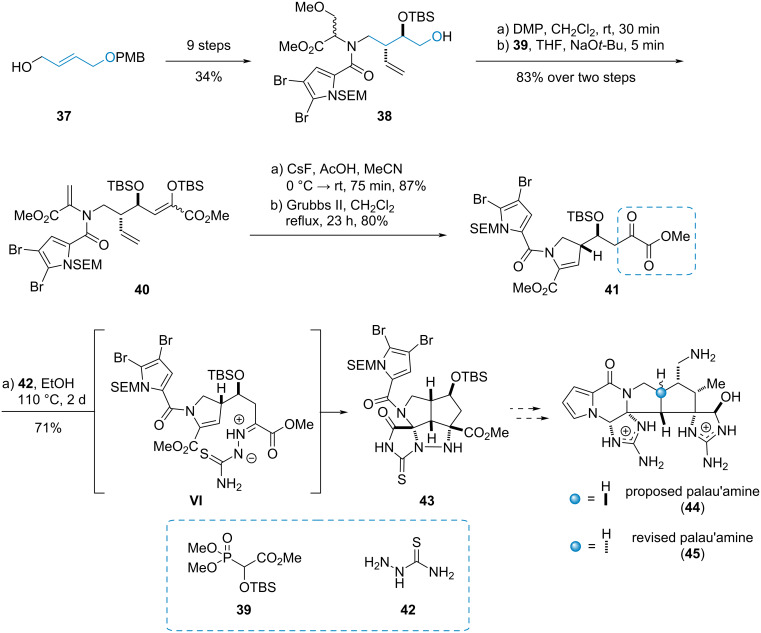
Azomethine imine cycloaddition towards the synthesis of the proposed structure of palau’amine (**44**) [19].

Palau’amine (**45**), a dimeric pyrrole-imidazole-bisguanidine alkaloid, was first isolated from the marine sponge *Stylotella aurantium* in 1993 [16–17]. It received considerable attention from the synthetic community because of its broad range of biological activities and complex structure. In an early endeavour of L. Overman et al. in 1997 [18] towards the originally proposed structure of palau’amine (**44**), a [3 + 2]-dipolar cycloaddition of α-ketoester **41** and the thiosemicarbazide **42**-derived azomethine imine **VI** to the triazacyclopenta[*cd*]pentalene **43** was utilized as a key step (Scheme 7) [18–21].

The α-ketoester **41** was accessible from amide **38**, which in turn was obtained from allylic alcohol **37**. Oxidation and Horner–Wadsworth–Emmons reaction with phosphonate **39** delivered the silyl enol ether **40**, which was deprotected and cyclized via a Grubbs metathesis to α-ketoester **41**. Subsequent cycloaddition delivered the advanced intermediate **43** in an efficient and elegant way.

#### Jatropha-5,12-diene

**Scheme 8:**
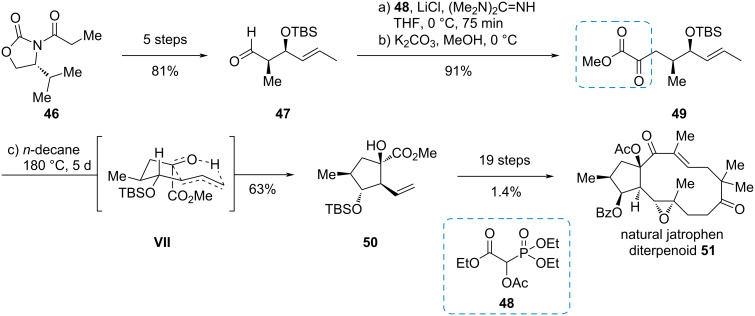
Intramolecular diastereoselective carbonyl-ene reaction of an α-ketoester in the synthesis of jatrophane diterpenoids [22].

Towards the total synthesis of natural and unnatural jatrophane diterpenes, Hiersemann et al. used a highly efficient, intramolecular carbonyl-ene reaction of α-ketoester **49** (Scheme 8) [22]. The ketoester was synthesized by a Horner–Wadsworth–Emmons reaction of phosphonate **48** with aldehyde **47**. Enantiopure aldehyde **47** was easily accessible from oxazolidinone **46** via Evans-aldol chemistry [23]. Heating of the α-ketoester **49** led to the highly substituted cyclopentanol **50** in a good dr of ≈5:1 (minor diastereomer not shown) via transition state **VII** where pseudo-1,3-strain is minimized. Nineteen further steps were necessary to give the naturally occurring jatrophen **51**.

#### (−)-Hopeanol

**Scheme 9:**
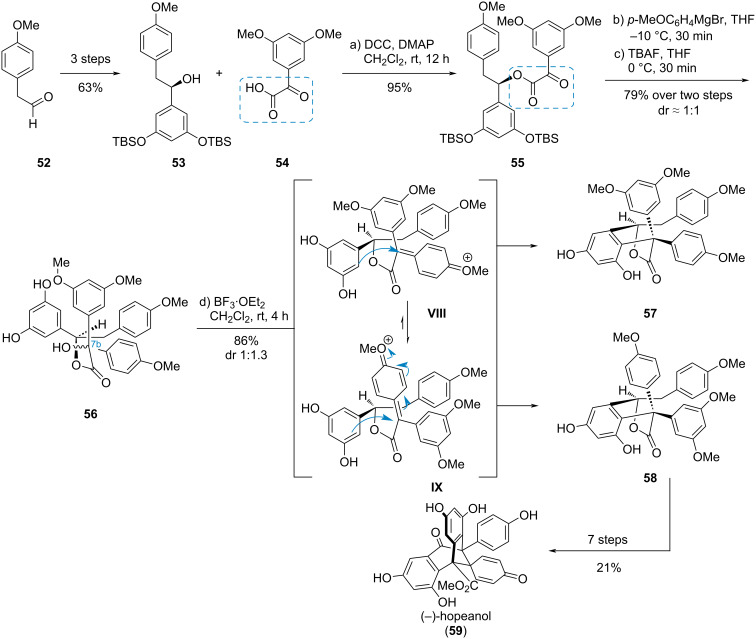
Grignard addition to an α-ketoester and subsequent Friedel–Crafts cyclization in the synthesis of (−)-hopeanol (**59**) [24].

In the synthesis of the polyphenolic natural product (−)-hopeanol (**59**), Nicolaou et al. used an α-ketoester moiety as a precursor for an intramolecular Friedel–Crafts cyclization (Scheme 9) [24]. Therefore, phenylacetaldehyde **52** was converted to the alcohol **53**, which was esterified with the α-ketoacid **54** to give ketoester **55**. Grignard addition to the keto carbonyl and subsequent TBS deprotection delivered the tertiary alcohol **56**, which was dehydroxylated to the diastereomeric cations **VIII** and **IX**. Friedel–Crafts reaction gave diastereomeric lactones **57** and **58**. The major diastereomer **58** could be converted to the complex polyphenol (−)-hopeanol (**59**) in seven further steps.

#### (+)-Camptothecin

**Scheme 10:**
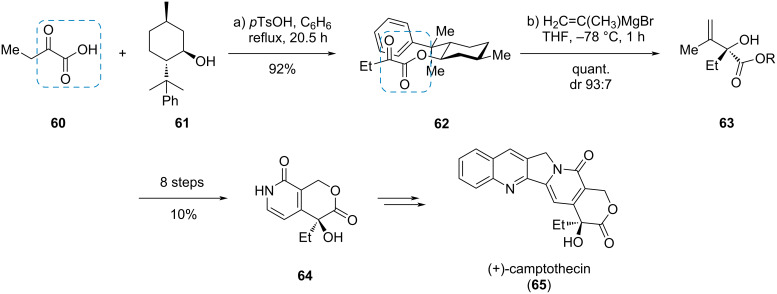
Diastereoselective addition to an auxiliary modified α-ketoester in the formal synthesis of (+)-campthotecin (**65**) [25].

In the formal synthesis of the pentacyclic, antiproliferative quinoline alkaloid camptothecin (**65**), Peters et al. used an α-ketoester moiety in an auxiliary controlled approach towards the only stereogenic center present in the natural product (Scheme 10) [25]. First, the ketoacid **60** was esterified with 8-phenylmenthol (**61**) to yield the α-ketoester **62**, followed by nucleophilic addition of isopropenylmagnesium bromide to give α-hydroxyester **63** in excellent yield and diastereoselectivity. Eight additional steps gave the bicyclic compound **64** which was already known from previous camptothecin syntheses.

#### Isoretronecanol

**Scheme 11:**
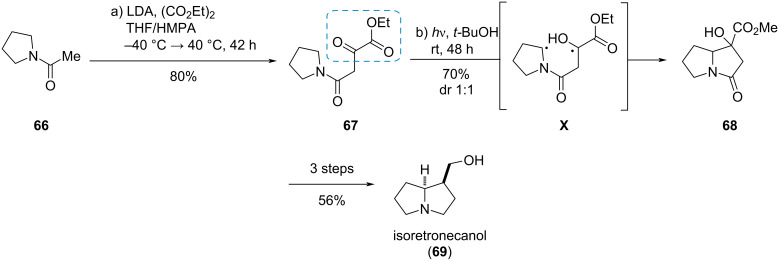
Intramolecular photoreduction of an α-ketoester in the synthesis of (*rac*)-isoretronecanol (**69**) [26].

The α-ketoester moiety can also be used in photochemical reactions, as shown by Gramain et al. in the synthesis of the pyrrolizidine alkaloid (*rac*)-isoretronecanol (**69**, Scheme 11) [26]. A Claisen condensation of the lithium enolate of *N*-acetylpyrrolidine (**66**) with diethyl oxalate gave the ketoester **67**. Irradiation of compound **67** with a medium pressure mercury lamp in Pyrex^®^ glassware triggered a 1,6-HAT leading to biradical **X** which combined to the racemic pyrrolizidine **68** as a 1:1 mixture of diastereomers. Three more steps gave the target compound **69** in 31% overall yield.

#### Corynoxine

**Scheme 12:**
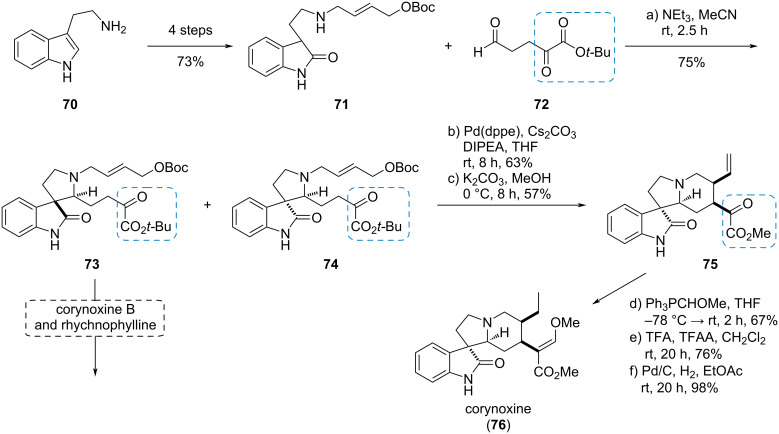
α-Ketoester as nucleophile in a Tsuji–Trost reaction in the synthesis of (*rac*)-corynoxine (**76**) [27].

Hiemstra et al. used the α-ketoester moiety for different purposes in the syntheses of a range of oxindole alkaloids. The start of the synthesis of (*rac*)-corynoxine (**76**) was the conversion of tryptamine (**70**) to oxindole **71**, which was used in a chemoselective Mannich reaction with aldehyde **72**, introducing the α-ketoester moiety (Scheme 12) [27].

The major *trans*-isomer **73** was further converted to the natural products corynoxine and rychnophylline. The minor *cis*-isomer **74** was used in an intramolecular Tsuji–Trost reaction, where the ketoester served as a nucleophile, which build up the piperidine ring and selectively set the desired *cis*-substitution. Subsequent transesterification gave the α-ketoester **75**, which was used in a Wittig reaction. The undesired *Z*-configured double bond was isomerized to the *E*-alkene and final hydrogenation delivered corynoxine (**76**).

#### (+)-Gracilamine

**Scheme 13:**
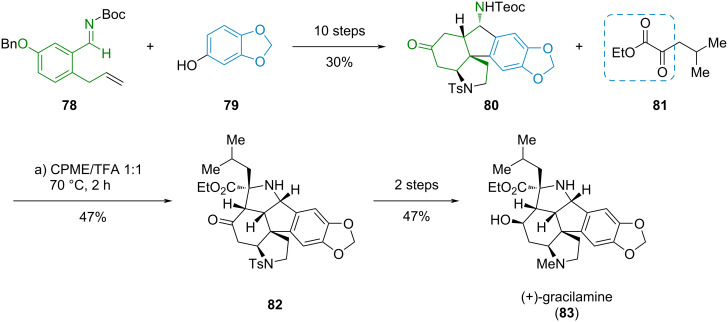
Mannich reaction of an α-ketoester in the synthesis of (+)-gracilamine (**83**) [28].

The Mannich reaction was also used by Nagasawa et al. as a key step in the synthesis of (+)-gracilamine (**83**), a pentacyclic alkaloid isolated from the plant *Galanthus gracilis*, (Scheme 13) [28]. The synthesis started from readily available sesamol (**79**) and imine **78** which gave the advanced intermediate **80** in ten steps. An intramolecular Mannich reaction of compound **80** with α-ketoester **81** furnished compound **82** with the last ring of the target (+)-gracilamine (**83**), which was accessible after two further steps.

#### (−)-Irofulven

**Scheme 14:**
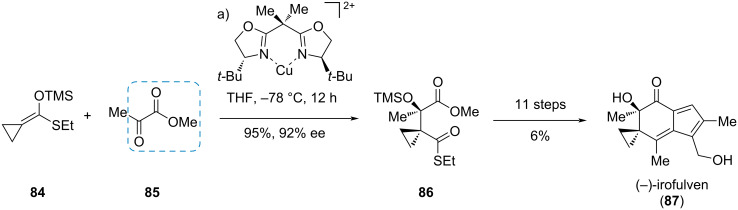
Enantioselective aldol reaction using an α-ketoester in the synthesis of (−)-irofulven (**87**) [29].

Irofulven (**87**) is a highly cytotoxic, semisynthetic drug obtained from the illudin sesquiterpene family. In a de novo synthesis towards (−)-irofulven (**87**), Movassaghi et al. used a Cu^II^-catalyzed asymmetric aldol reaction of *O*-silyl ketene *S*,*O*-acetal **84** with methyl pyruvate (**85**) to enantioselectively install the crucial tertiary TMS-protected alcohol in ester **86** (Scheme 14) [29]. Eleven further steps gave (−)-irofulven (**87**).

### 2. Mesoxalic diesters and ester amides as key intermediates

#### (+)-Awajanomycin

**Scheme 15:**
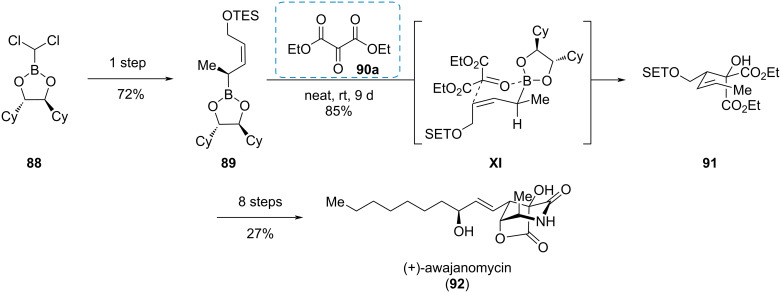
Allylboration of a mesoxalic acid ester in the synthesis of (+)-awajanomycin (**92**) [30–31].

Diethyl mesoxalate (**90a**) is a valuable building block due to the high density of carbon atoms in high oxidation states. As a *vic*-tricarbonyl compound, its central keto group is an especially potent electrophile. The Koert group used this reactivity in their synthesis of (+)-awajanomycin (**92**), a marine natural product with a γ-lactone-δ-lactam core structure (Scheme 15) [30–31]. Key step was an asymmetric allylboration of diethyl mesoxalate (**90a**) with boronate **89**, which was easily accessible through a Matteson homologation of dichloromethyl boronate **88**. The reaction of (*Z*)-alkenyl boronate **89** with mesoxalate **90a** delivered product **91** through the six-membered transition state **XI**. Eight further steps accomplished the total synthesis of (+)-awajanomycin (**92**).

#### (−)-Aplaminal

**Scheme 16:**
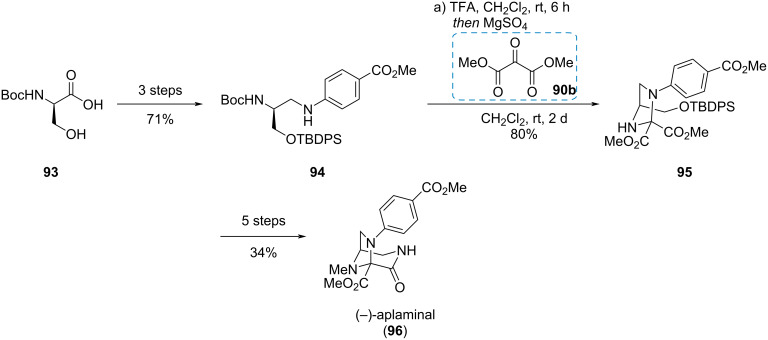
Condensation of a diamine with mesoxolate in the synthesis of (−)-aplaminal (**96**) [32].

Dimethyl mesoxalate (**90b**) was used by Smith and Liu in the synthesis of the cytotoxic metabolite (−)-aplaminal (**96**), which was isolated from the sea hare *Aplysia kurodai* [32]. The natural product is characterized by a triazabicyclo[3.2.1]octane, where each bridge possesses a nitrogen atom. The synthesis commenced with *N*-Boc-serine (**93**) which was converted to secondary aniline **94** in three steps (Scheme 16). Subsequent deprotection and condensation with dimethyl mesoxalate (**90b**) gave imidazolidine **95**. With compound **95** at hands, five further steps gave (−)-aplaminal (**96**) in a good overall yield of 19%.

#### Cladoniamide G

**Scheme 17:**
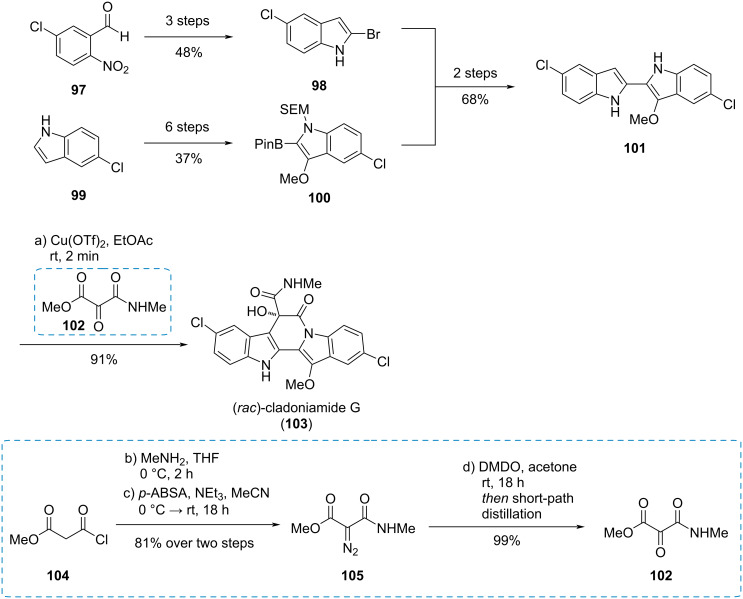
Synthesis of mesoxalic ester amide **102** and its use in the synthesis of (*rac*)-cladoniamide G (**103**) [33].

The unsymmetrical mesoxalic acid amide **102** was used by Koert et al. in the racemic synthesis of the bisindole alkaloid (*rac*)-cladoniamide G (**103**, Scheme 17) [33]. The synthesis started with benzaldehyde **97** and indole **99** which were converted to the indole building blocks **98** and **100**, respectively. These were connected to bisindole **101**, which reacted with mesoxalic ester amide **102** in a Friedel–Crafts reaction followed by a spontaneous lactamization to give (*rac*)-cladoniamide G (**103**). The mesoxalic ester amide **102** was synthesized from malonyl chloride **104** through amidation and Regitz diazotransfer, yielding diazo compound **105**. Subsequent oxidation and dehydration of the resulting hydrate through short-patch distillation gave the desired *vic*-tricarbonyl compound **102**.

### 3. α,β-Diketoesters as key intermediates

#### Preussochromone E and F

**Scheme 18:**
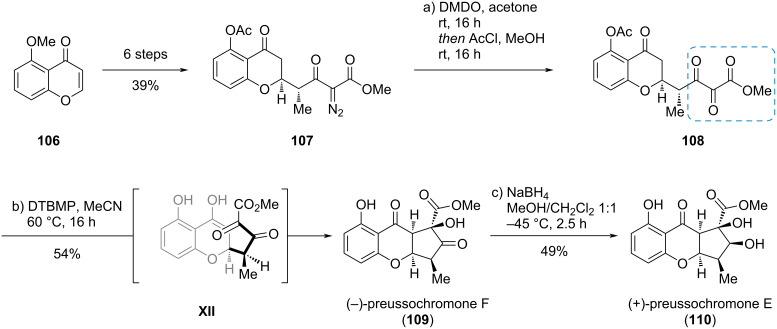
The thermodynamically controlled, intramolecular aldol addition of a *vic*-tricarbonyl compound in the synthesis of preussochromones E (**110**) and F (**109**) [34].

In a short and enantioselective total synthesis of preussochromone E (**110**) and F (**109**), Koert et al. used the complex *vic*-tricarbonyl compound **108** to set two stereogenic centers and correct one via an intramolecular aldol addition (**108** → **109**; Scheme 18) [34]. The *vic*-tricarbonyl compound **108** was synthesized via DMDO oxidation from α-diazo-β-ketoester **107**, which was easily accessible from 5-methoxy-4*H*-chromen-4-one (**106**). The thermodynamically controlled basic intramolecular aldol addition of compound **108** using the bulky amine base 2,6-di-*tert*-butyl-4-methylpyridine (DTBMP) led to epimerization of the methyl group and cyclization, giving preussochromone F (**109**) as single isolable diastereomer probably via transition state **XII**. The subsequent reduction of compound **109** gave preussochromone E (**110**).

## Conclusion

The variety of examples prove that vicinal ketoesters are valuable synthetic intermediates for the synthesis of complex target structures such as natural products. α-Ketoesters, mesoxalic esters, and α,β-diketoesters can be used bearing an electrophilic keto group as reactive site. The vicinal arrangement of carbonyl groups allows the stabilization of reactive conformations by chelation or dipole control. Suitable key reactions are e.g., aldol additions, carbonyl ene reactions, Mannich reactions, and additions of organometallic reagents. The presented examples may encourage the use of vicinal ketoesters in future applications, in particular in the field of natural product synthesis.
